# Solution phase treatments of Sb_2_Se_3_ heterojunction photocathodes for improved water splitting performance†

**DOI:** 10.1039/d3ta00554b

**Published:** 2023-03-21

**Authors:** Pardis Adams, Fabrizio Creazzo, Thomas Moehl, Rowena Crockett, Peng Zeng, Zbynek Novotny, Sandra Luber, Wooseok Yang, S. David Tilley

**Affiliations:** a Department of Chemistry, University of Zurich Zurich Switzerland david.tilley@chem.uzh.ch; b Advanced Material and Surfaces, EMPA Dubendorf Switzerland; c Scientific Centre for Optical and Electron Microscopy (ScopeM), ETH Zurich Switzerland; d Swiss Light Source, Paul Scherrer Institute Villigen-PSI Switzerland; e Laboratory for Joining Technologies and Corrosion, EMPA Dübendorf Switzerland; f School of Chemical Engineering, Sungkyunkwan University (SKKU) Suwon South Korea wooseok.yang@skku.edu; g SKKU Institute of Energy Science and Technology (SIEST), Sungkyunkwan University Suwon 16419 Republic of Korea

## Abstract

Antimony selenide (Sb_2_Se_3_) is an auspicious material for solar energy conversion that has seen rapid improvement over the past ten years, but the photovoltage deficit remains a challenge. Here, simple and low-temperature treatments of the p–n heterojunction interface of Sb_2_Se_3_/TiO_2_-based photocathodes for photoelectrochemical water splitting were explored to address this challenge. The FTO/Ti/Au/Sb_2_Se_3_ (substrate configuration) stack was treated with (NH_4_)_2_S as an etching solution, followed by CuCl_2_ treatment prior to deposition of the TiO_2_ by atomic layer deposition. The different treatments show different mechanisms of action compared to similar reported treatments of the back Au/Sb_2_Se_3_ interface in superstrate configuration solar cells. These treatments collectively increased the onset potential from 0.14 V to 0.28 V *vs.* reversible hydrogen electrode (RHE) and the photocurrent from 13 mA cm^−2^ to 18 mA cm^−2^ at 0 V *vs.* RHE as compared to the untreated Sb_2_Se_3_ films. From SEM and XPS studies, it is clear that the etching treatment induces a morphological change and removes the surface Sb_2_O_3_ layer, which eliminates the Fermi-level pinning that the oxide layer generates. CuCl_2_ further enhances the performance due to the passivation of the surface defects, as supported by density functional theory molecular dynamics (DFT-MD) calculations, improving charge separation at the interface. The simple and low-cost semiconductor synthesis method combined with these facile, low-temperature treatments further increases the practical potential of Sb_2_Se_3_ for large-scale water splitting.

## Abstract 2

The PEC performance of antimony selenide (Sb_2_Se_3_) is increased by removing oxide impurities from the surface using (NH_4_)_2_S etching solution and passivating dangling bonds with a CuCl_2_ solution treatment.

## Graphical abstract

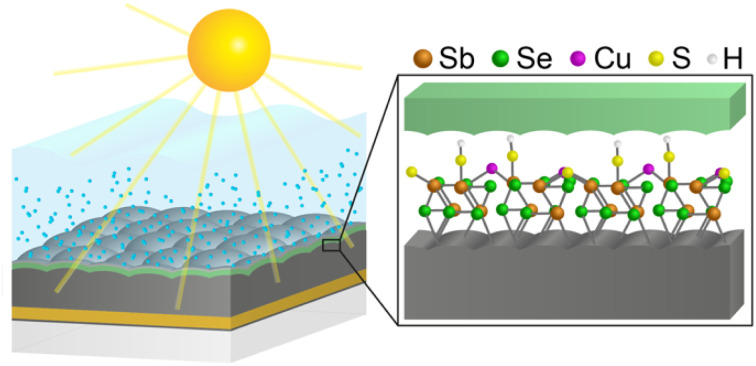

## 1. Introduction

Meeting the increasing energy demand of today's world whilst attempting to achieve net zero emissions is one of the top priorities of this century. The sun's ample energy availability makes it a potential candidate to meet such demand in a green and sustainable manner.^1^ Since solar-related energy sources are subject to weather fluctuations and the diurnal cycles, a practical solution is to store the energy in molecular bonds, as nature does through photosynthesis, such as those of H_2_, which can then be used as a fuel source. Therefore, the semiconductor-based conversion of solar energy to H_2_ by water splitting is advantageous for our already fuel-based economy to directly convert and store the energy within the same system.^2–5^ The constraints on photoelectrochemical (PEC) water-splitting cells arise from three fundamental system requirements: efficient photogenerated charge collection, harvesting a substantial fraction of the available solar energy, and long-term stability. Due to the inherent area-dependency and hence the amount of material used, the predominant way to make PEC cells more competitive is by increasing their solar to hydrogen (STH) efficiency.^6^ Sb_2_Se_3_ has recently gained popularity due to its potential to have similar STH efficiencies to crystalline silicon, the benchmark material for solar conversion technology. Sb_2_Se_3_ is a relatively cost-effective (Sb has a similar cost to Cu and Se has a similar cost to Sn) and reasonably abundant light-absorbing material, not listed as a “highly toxic” compound in many official records, as opposed to many other current semiconductor materials containing Cd and Pb.^7^ The melting point of Sb_2_Se_3_ lies at 608 °C, making it suitable for low-temperature processing and easy deposition. The Sb_2_Se_3_ polycrystalline films have no other stable binary phase and a quasi-one-dimensional (Q1D) crystal structure oriented along the [001] direction.^8^ As opposed to other widely explored 3D photovoltaic absorbers such as CIGS and GaAs, the Q1D Sb_2_Se_3_ has intrinsically benign grain boundaries (GB) along the ribbons and indeed at any crystallographic orientation due to the “self-healing” effect,^9,10^ hence avoiding major surface recombination losses that limit high-efficiency thin-films. As a light absorber material for solar cells, Sb_2_Se_3_ was first reported in 2014. Since then, the conversion efficiency has rapidly increased from 2.26% to 9.2% and recently to over 10.0% when alloyed with sulphur (Sb_2_(S, Se)_3_).^11–13^ Even though within the PEC world, efficiencies are still low, with its optimal band gap at ∼1.2 eV high absorption coefficient (>10^5^ cm^−1^ in the ultraviolet and visible spectrum) and resistance to photo corrosion, Sb_2_Se_3_ is a prime candidate as a photoabsorber material for solar water splitting.^14–16^ A key challenge currently limiting the device performance is the low open circuit voltage (*V*_oc_), mainly attributed to Sb_Se_ antisite defects with mid-gap transition levels that can act as recombination centres.^17–19^ There are several common approaches to tackle this complex problem, such as the passivation of surface states by optimisation of the device structure considering the lattice matching between layers,^20^ an adaptation of the carrier extraction selectivity at the interface,^17^ and modification of the band alignment.^21^

In this study, the focus was placed on increasing the onset potential of the Sb_2_Se_3_ photocathode by interfacial engineering, more precisely through surface modification of the photoactive material.^22^ It is well-known that at the semiconductor interface, the presence of surface states acts as trapping sites and recombination centres.^23,24^ Therefore, simple, low-cost, and scalable solution-based treatments using earth-abundant chemicals with comparatively low toxicities were used in this study to passivate the above-mentioned surface states and, in turn, have better charge separation and reduced recombination. Several etchants have proven effective in removing the oxide layer on the Sb_2_Se_3_ surface, of which (NH_4_)_2_S was chosen as the most promising due to its very low toxicity, good solubility in water and high etching rates without requiring additional heating.^23^ As observed in other chalcogenides such as GaAs and Cu(In,Ga)Se_2_,^23,24^ (NH_4_)_2_S is known to remove carbon impurities as well as the top oxide layer, which is often created as a result of dangling bonds' exposure to air at high temperatures during the selenisation process and thereafter until the overlayer is deposited. It could also incorporate sulphur onto the surface, which then passivates the dangling bonds on the surface.^24,25^ CuCl_2_ has been shown to have an effect on the performance of Sb_2_Se_3_ solar cells in a superstrate configuration (*i.e.* by modifying the Sb_2_Se_3_/Au interface); however, the reason for this improvement is debated.^26^ Therefore, we have undertaken a comprehensive study of different metal and halogen combinations as controls to elucidate the role of the CuCl_2_ treatment and the (NH_4_)_2_S etching treatment. Density functional theory molecular dynamics (DFT-MD) was performed, finding a good agreement with the experimental observations, *i.e.*, Cu is binding to selenium atoms and passivating the surface states, therefore removing recombination centres and improving charge separation at the interface of the Sb_2_Se_3_ and TiO_2_, which lead to the enhanced performance.

## 2. Results and discussion

**Fig. 1:**
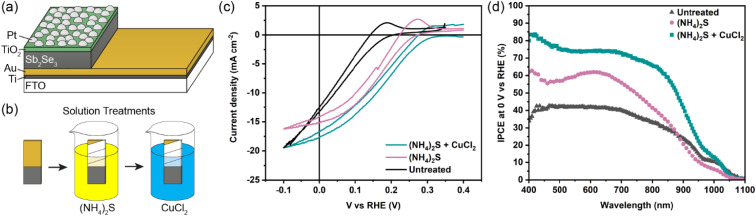
(a) A schematic showing the device configuration of Sb_2_Se_3_ photocathodes. All samples have an FTO/Ti/Au/Sb_2_Se_3_/TiO_2_/Pt configuration. The difference lies in the treatments undertaken before the TiO_2_/Pt layer deposition. (b) A schematic showing the (NH_4_)_2_S and CuCl_2_ treatment processes. Before the TiO_2_/Pt layers were deposited, the samples were wrapped in Teflon tape and etched/treated sequentially with (NH_4_)_2_S and CuCl_2_ solutions. (c) Current density *vs.* voltage plots of a typical sample in 1 M H_2_SO_4_ under simulated 1 sun illumination. (d) Quantum efficiency spectra in 1 M H_2_SO_4_ at 0 V *vs.* RHE under 1% sun-white light bias.

**Fig. 2:**
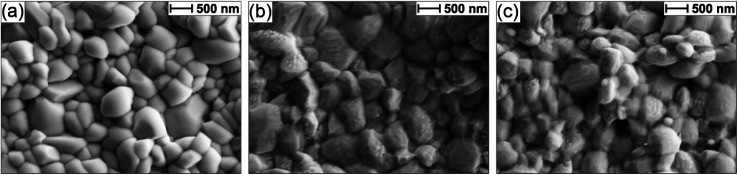
SEM images for an FTO/Ti/Au/Sb_2_Se_3_ sample (a) untreated, (b) etched *via* (NH_4_)_2_S, and (c) etched *via* (NH_4_)_2_S + CuCl_2_ treatment.

**Fig. 3:**
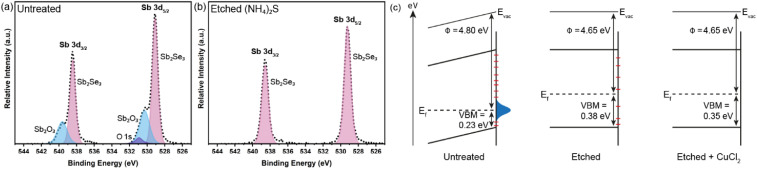
Synchrotron-based XPS core level spectra of Sb 3d for (a) untreated Sb_2_Se_3_ and (b) Sb_2_Se_3_ etched with (NH_4_)_2_S. (c) Band alignment changes due to the Sb_2_O_3_ layer (represented by the blue Gaussian) and surface states (represented by red dashes), measured by KP-FM and XPS valence band maxima (VBM). Band bending is represented as a sloping line due to the low doping (the space charge region is larger than the film thickness).

The Sb_2_Se_3_ compact films benefiting from high performances in this study were prepared by the selenisation of electrodeposited Sb films.^14^ The film composition and structure in Fig. 1a are the basis of all films discussed in this study. Ti is used as a conductive anchoring layer to allow for better adhesion of the Au layer to the FTO substrate. The Au layer acts *via* an ohmic contact formation to the photo absorber as the hole-extracting contact. The photo absorber layer of Sb_2_Se_3_ is then covered by a TiO_2_ layer deposited by atomic layer deposition (ALD). The TiO_2_ layer not only protects the active layer from eventual corrosion in the acidic media of the electrolyte but also forms a p–n junction with the photo absorber allowing for better charge separation. Finally, the Pt acts as a catalyst for the hydrogen evolution reaction (HER), facilitating enhanced charge transfer. With Fig. 1b, the experimental procedure of the etching and CuCl_2_ treatment can be visualised. A large improvement in the performance of these films arises from the etching of the Sb_2_Se_3_ surface with (NH_4_)_2_S solution, as well as treating the etched surface with a CuCl_2_ solution. Each step of this procedure enhances both the photovoltage and photocurrent of the thin film. This increase can be observed in the cyclic voltammetry plots in Fig. 1c. Multiple CV experiments were undertaken to ensure reproducibility. With the statistical plot of multiple samples, the same increasing trend is observed, as seen in Fig. S1.† Whilst the untreated film gives ∼13 mA cm^−2^ at 0 V *vs.* reversible hydrogen electrode (RHE) with an onset potential of 0.14 V *vs.* RHE, the etched film shows an increased photovoltage of 0.22 V *vs.* RHE and a photocurrent of 15 mA cm^−2^ at 0 V *vs.* RHE. The CuCl_2_-treated film is further improved with a photovoltage of 0.28 V *vs.* RHE and a photocurrent of 18 mA cm^−2^ at 0 *vs.* RHE. The incident photon to current efficiency (IPCE) spectrum of the Sb_2_Se_3_ after etching and CuCl_2_ treatment shows a stepwise increase in the quantum efficiency, increasing from 42% to 62% and further to 74% at 600 nm for the untreated, etched and etched + CuCl_2_ treated films respectively (Fig. 1d). Integrations of the IPCE data were also obtained and plotted in Fig. S2a.† Fig S2b,† is an example of a typical (NH_4_)_2_S + CuCl_2_ sample which clearly shows that the performance of the device is not directly proportional to the intensity of the light. Whilst IPCE is measured at ∼1% sun (with the white light bias), the CV measurements are obtained at 100% sun and suffer from additional recombination losses at these higher light intensities. This light dependency can vary from sample to sample and is the reason for the discrepancy between the integrated current data and CV measurements in Fig. 1c. Stability measurements at 0.1 V *vs.* RHE in Fig. S2c† indicate a robust improvement of the treated samples and stability of over 4 hours. The decrease in stability likely results from the Pt catalyst detachment as after redeposition of Pt, most of the current is revived. Various control experiments were performed to understand the performance improvements' origin. These comparisons were undertaken with samples synthesised in the same batch so that any synthesis-to-synthesis variations could be eliminated. First, as seen in Fig. S3a,† the order of the treatments was reversed, such that films were first treated with CuCl_2_ and then etched with (NH_4_)_2_S, resulting in lower photovoltage and photocurrent than the champion CuCl_2_ treated sample of that batch (though still improved *versus* the untreated film). The performance of this reversed order sample is similar to the (NH_4_)_2_S treated sample without CuCl_2_ treatment, implying that the CuCl_2_ treatment is only effective after the etching process (blue curve in Fig. S3a†). It is hypothesised that this is due to Cu binding to the Se dangling bonds on the surface, which are absent before the etching process due to the surface Sb_2_O_3_ layer (*vide infra*). In addition, an annealing treatment (at 200 °C for 20 minutes) after the CuCl_2_ treatment was investigated as it may facilitate the incorporation of CuCl_2_ into lattice sites of the Sb_2_Se_3_ film. This experiment showed that further annealing does not increase the performance, meaning that the activation energy of the CuCl_2_ treatment is low and likely does not involve substitution at a lattice site (Fig. S3a,† red CV curve). Also, other chloride and copper sources were tested *via* other metal salts (MgCl_2_, CsCl and Cu(NO_3_)_2_). The solutions were produced with the same concentration and in the same solvent to minimise the effects of external factors. Fig. S3b† shows that whilst the MgCl_2_ in ammonia solution precipitated to produce Mg(OH)_2_ and hence had an entirely different and even detrimental effect on the sample, the CsCl showed almost no difference as compared to the untreated sample of that batch. On the other hand, Cu(NO_3_)_2_ revealed an effect similar to CuCl_2_ and within the range of error. The experiments showed that although Cu generally influences the performance of the photocathode, Cl does not affect the performance at all. Fig. 2a highlights morphological differences between the three samples *via* scanning electron microscopy (SEM). It shows that whilst the untreated sample has smooth and homogenous grain surfaces, the etched sample (Fig. 2b) is visibly roughened. The etched + CuCl_2_ treated sample (Fig. 2c), however, shows a similar morphological composition to the etched samples, which is in line with the fact that the Cu has negligible structural effects as it only targets the topmost Se atoms. This can also be observed in images obtained from atomic force microscopy-Kelvin probe force microscopy (KP-FM) in Fig. S4.† X-ray diffraction (XRD) measurements revealed a (*hkl*) preferential orientation among all samples. The XRD data also indicate that there are no significant differences (*i.e.*, changes in lattice spacing or preferential orientation) between the three different samples (Fig. S5†). To further understand the changes incurred by the different treatments, core levels of the main elements in the samples were measured by X-ray photoelectron spectroscopy (XPS). A significant change was observed in the Sb core level before and after the etching treatment (Fig. 3a and b). The higher binding energy shoulders observed around 530 eV and 540 eV denoted as Sb_2_O_3,_ as well as the oxygen 1s peak at 532 eV, indicate surface oxidation due to atmospheric exposure of the dangling bonds of the bare Sb_2_Se_3_ film after fabrication. This phenomenon has been observed in other studies, but whilst the oxide layer is deemed beneficial in the PV cells with a superstrate configuration (where the Sb_2_O_3_ acts as a buffer layer at the Sb_2_Se_3_/Au interface, making the contact more selective for holes); this layer has a detrimental effect on the substrate configurations.^27,28^ The Sb_2_O_3_ induces a Fermi-level pinning effect, causing an unfavourable surface band bending (Fig. 3c, drawn without curvature due to the near intrinsic level of doping in the semiconductor and consequently the very wide space charge region). This phenomenon makes electron transfer across the interface unfavourable and decreases the photogenerated charges' lifetime. The Fermi level position was measured by XPS valence band maxima and KP-FM measurements (Fig. S4 and S6†). After removing the oxide layer by etching, a decrease in the work function is observed, which remains unchanged (within the experimental error) following the CuCl_2_ treatment. The similar valence band maxima (VBM) measured by XPS indicates that the Cu does not dope the semiconductor and only acts to passivate the surface states. As seen in Fig. S7,† diffuse reflectance spectroscopy (DRS) confirms that the band gap of the Sb_2_Se_3_ remains the same after each treatment, which is in agreement with the band diagram in Fig. 3c and indicates that the treatments only target the surface of the samples.

**Fig. 4:**
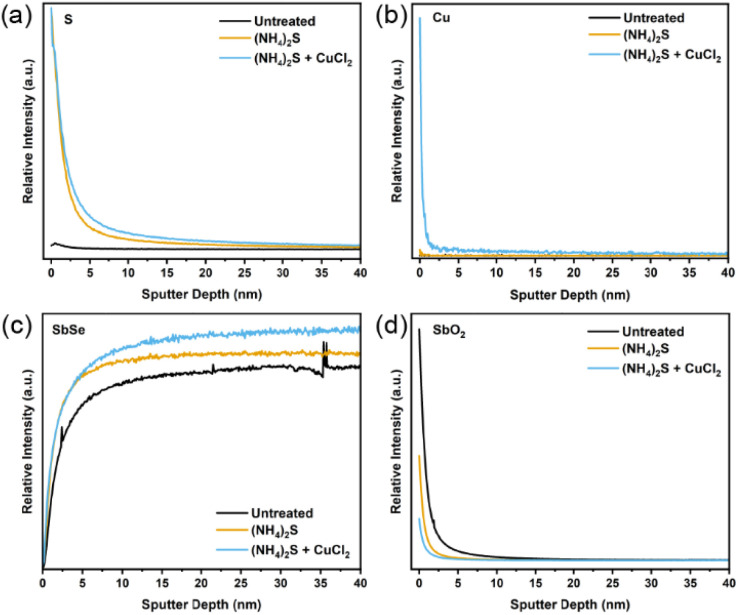
ToF-SIMS sputter depth profile for (a) sulphur, (b) copper, (c) antimony selenide, (d) antimony oxide.

Transmission electron microscopy (TEM) was used to closely observe the grains and grain boundaries for the three sample types. Fig. S8–S10† clearly show the distribution of elements, local structure, and crystallinity of different layers in these devices. However, no significant differences were observed between the three samples. EDX measurements following TEM were also undertaken in order to detect S or Cu near the surface or in the grain boundaries. Again, no significant differences were obtained from the EDX data. In Fig. S11,† a line scan passing through two Sb_2_Se_3_ grains and a grain boundary revealed a striking homogeneity of Sb, Se, Cu and Cl across grain boundaries, indicating that the amount of each element does not vary significantly throughout. This suggests that Cu does not tend to accumulate within the grain boundaries, contrary to the previous study showing the grain boundary inversion effect by the CuCl_2_ treatment on Sb_2_Se_3_.^26^ To detect the low amount of S and Cu on the surface, time of flight mass spectrometry (ToF-SIMS) was employed. From Fig. 4a, a substantial S signal is observed after the etching step and remains on the surface even after the CuCl_2_ treatment. The Cu content also increases after the CuCl_2_ treatment, as seen in Fig. 4b. Some Cu penetrates even further into the film; whilst this may be due to experimental noise, it may also be indicative of an exceedingly small amount of Cu making a bond with sublayer atoms. This behaviour is seen during DFT simulations and will be explained further in the next section. Both the S and the Cu content are highest at the surface. This coincides well with the lower amount of Sb and Se on the surface as opposed to the bulk (Fig. 4c). Similarly, from Fig. 4d, it can be observed that the CuCl_2_ treatment prevents the reoxidation of the photo absorber surface, allowing for a better interface with TiO_2_ as antimony oxide amounts on the surface also decrease and remain low after the etching treatment. Chloride content appears in all three films, which indicates that it may be present as a contaminant in the precursors from which the Sb_2_Se_3_ is synthesised or from exposure to air prior to the measurement (Fig. S12†). Regardless, as chlorine has not been detected by any other methods and does not appear to have any effect when films are treated with other Cl sources, it can be concluded that Cl does not bind with or otherwise integrate into Sb_2_Se_3_. This is in agreement with the literature in which chlorine is known to have a high formation enthalpy for interstitial doping, and the temperatures in this treatment are insufficient for the substitutional doping of this element into the Sb_2_Se_3_ thin films.^19^ Previous experimental studies also show that Cl ions tend to stay at the surface and not diffuse into the film. This is likely due to their large ion radius of 1.81 Å and electrostatic repulsive force, which can account for their negligible effects on device performance.

**Fig. 5:**
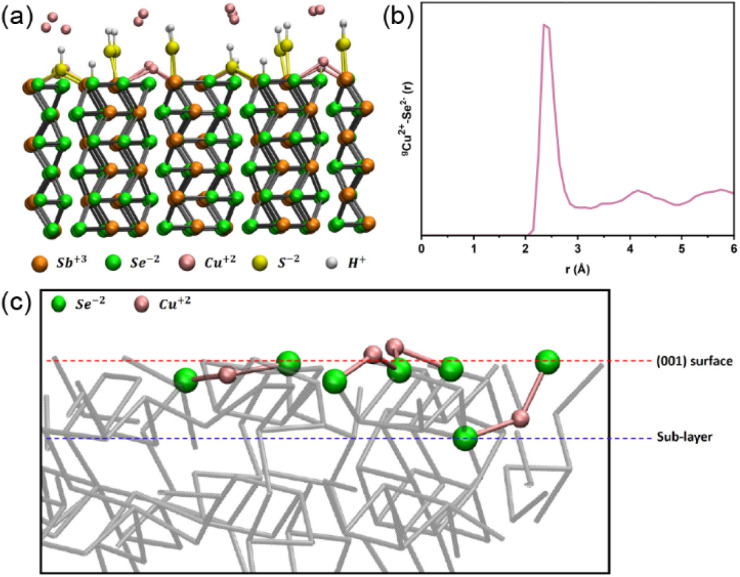
(a) Zoom-in view of the simulation box: (001) Sb_2_Se_3_ at the interface with 16 Cu atoms. Each Sb surface atom is terminated by S–H, and some Se surface atoms are terminated by hydrogen atoms (see text for details). Antimony atoms are presented in orange, and selenium atoms are in green. Sulphur atoms in yellow and hydrogen in white colour, respectively. (b) Radial distribution function between surface Se atoms and Cu ions. (c) Average position of Cu ions (in pink colour) during the simulation time.

Spin-polarised DFT-MD was employed to shed light on the mechanism of action of the treatments. The (001) Sb_2_Se_3_ surface was chosen as a suitable simplified representation of the surface of the polycrystalline films due to the preferential (*hkl*) orientation observed by XRD. The (001) Sb_2_Se_3_ crystal was modelled where the ribbons had been cut, resulting in dangling bonds (Fig. 5a). 16 Cu ions were placed at the interface with the (001) Sb_2_Se_3,_ and DFT-MD was performed at a temperature of 150 °C, which is the maximum temperature of the ALD chamber; the next synthesis step of these thin films. Analysing a 40 ps long DFT-MD trajectory, it was found that all the interfacial Cu ions moved towards the surface, making covalent bonds with the (001) Sb_2_Se_3_ surface. In particular, all Cu ions are surface adsorbed, making (on average) 2 bonds with Se surface atoms at a distance of 2.3 Å as highlighted by the first peak position of the radial distribution function between surface Se atoms and Cu ions in Fig. 5b. Cu ions become coordinatively saturated once adsorbed at the surface, finding their ‘equilibrium’ position.

The temperature of 150 °C does not seem to be enough to break Cu–Se surface bonds and drive Cu ions into the Sb_2_Se_3_ structure (channels). The average position of the bound Cu ions during the DFT-MD simulation has been found at the level of the (001) Sb_2_Se_3_ surface, which involves a local distortion into the regular crystalline geometry of the (001)-surface pattern. However, during the DFT-MD, only one Cu atom (out of the 16 Cu atoms placed initially at the interface) positions itself between the (001) surface and the sublayer of the solid structure, evidencing a slightly more penetrating behaviour (binding to one surface Se atom and one sublayer Se atom) as shown in Fig. 5c. Although this could be ascribed to Cu-motion (‘fluctuations’) around the equilibrium position at the surface, it would be in line with the ToF-SIMS measurements where Cu atoms have been observed in the outermost layers of the slab. The calculation showed that Cu ions could bind to the Se ions at the (001) surface of Sb_2_Se_3_. Together with the experimental observations, in which the performance was enhanced by the CuCl_2_ treatment with favourable band bending, it implies that forming the Cu–Se bonds can passivate the detrimental surface states.

## 3. Experimental

### 3.1 Synthesis of Sb_2_Se_3_ photocathode

The FTO TEC 15 substrates from Pilkington were cleaned by consecutive washing in water and ethanol in an ultrasonic bath and treated for 30 minutes under UV/ozone to remove surface contaminants. 10 nm of Ti adhesion layer and 150 nm of Au were coated onto the FTO substrates using a Safematic CCU-010 sputter coater. In a three-electrode configuration, Sb metal was electrodeposited on top of the FTO/Ti/Au substrates. The Sb solution (100 mL) consisting of 15 mM potassium antimony tartrate K_2_Sb_2_(C_4_H_2_O_6_)_2_ (Sigma-Aldrich, ≥99%) and 50 mM tartaric acid (Acros Organics, 99.5%) was adjusted to a pH of 1.3 (pH adjusted using conc. sulfuric acid, (Sigma-Aldrich, ≥99%)). To electrodeposit the Sb, a potential of −0.3 V *vs.* Ag/AgCl was applied, and the thickness of the Sb metal was controlled by limiting the charge passed to 1.4 C cm^−2^.^14,29^ A two-zone furnace was used to selenise the electrodeposited Sb substrate. 30 mg of selenium pellets were placed symmetrically around the Sb substrate, and the chamber was purged with argon. A ramping rate of 15 °C min^−1^ was used to reach 350 °C, and the sample was held at this temperature for 40 min under static argon. After this 60 minute procedure, the furnace lid was opened to cool down the tube to room temperature, requiring approximately 60 minutes. After the selenisation, the exposed area of the Au layer (*i.e.*, where Sb was not deposited) remains the same colour and is still conductive, indicating that the Au layer is stable during this process. This simple synthesis method enables the fabrication of high-quality compact thin films without requiring sophisticated high vacuum equipment. However, small leaks in the gas connection to the tube may cause a Sb_2_O_3_ layer to form on top of the Sb_2_Se_3_. Also, the selenisation process converts Sb metal to Sb_2_Se_3_, and therefore some unreacted Sb metal may remain in the final photoabsorber layer. This largely differs from other methods directly using a Sb_2_Se_3_ precursor. When using this electrodeposition-based method, the designated deposition area must be closely monitored in order to have reproducible thickness and performance. A 100 nm thick layer of TiO_2_ was deposited *via* atomic layer deposition (ALD) using a Picosun R200 system. Ti and O were sourced from tetrakis(dimethylamido)titanium (TDMAT), and H_2_O. The temperature of the TDMAT precursor cylinder was held at 85 °C, and the reactor temperature at 120 °C. The thickness of the TiO_2_ layer was confirmed by ellipsometry on a silicon witness wafer. In a final step, a nominally 2 nm thick layer of Pt was sputtered onto the photocathode.

### 3.2 Solution treatments

The exposed Au surface was protected with Teflon tape before all treatments. The Sb_2_Se_3_ thin films were dipped for 5 s into a clear yellow aqueous (NH_4_)_2_S solution (Sigma-Aldrich, 40–48 wt% in H_2_O) (10 mL, 10–12 wt%) at room temperature, then rinsed for 10 s with distilled water and dried under N_2_ flow. The CuCl_2_ treatment was undertaken following the etching procedure. Films were dipped for 5 min into a clear dark blue aqueous CuCl_2_ (Aldrich, 99%, powder) solution (10 mM in 28–30% in aqueous NH_3_) at room temperature. Subsequently, the samples were rinsed with distilled water for 10 s and dried under N_2_ flow. The MgCl_2_ (Sigma-Aldrich, anhydrous for synthesis), CsCl (Sigma-Aldrich, 99.9%) and Cu(NO_3_)_2_ (Merck, Supelco, for analysis) solutions used for control experiments were prepared with the same concentration and solvent as the CuCl_2_ solution (10 mM in 28–30% aqueous NH_3_). These treatments were likewise applied following the (NH_4_)_2_S etching step. As evident in Fig. S3a,† this quick dipping treatment at room temperature does not require further annealing or other energy-intensive equipment. The treatment solutions are also aqueous and non-toxic (as opposed to other etchants such as HF), making them simple to prepare and handle.

### 3.3 Photoelectrochemical (PEC) characterisation of Sb_2_Se_3_

The photoelectrochemical performance of the photocathodes was measured in a three-electrode configuration using a BioLogic SP-200 potentiostat under irradiation from simulated AM 1.5G illumination, calibrated using a silicon diode from PV Measurements, Inc. (100 mW cm^2^). A 1 M H_2_SO_4_ (pH 0) solution was used for the measurements. The three-electrode configuration consisted of an Ag/AgCl (3 M KCl) reference electrode, a freshly cleaned Pt wire as the counter electrode and the photocathode as the working electrode. The CV was always measured with a scan speed of 10 mV s^−1^ and scans were performed from positive to negative to positive potential. The photocathode area was defined by epoxying (Loctite 9461) around an O-ring (ID 7 mm) that was placed on the sample surface (Fig. S13†). IPCE was measured on a home built IPCE system featuring a halogen light source with a double monochromator and a white light bias from LED. The IPCE was measured in a three-electrode configuration as above at 0 V *vs.* RHE at 5 nm wavelength intervals and 1% white light bias.

### 3.4 Morphology and crystal characterisation

The plan view scanning electron microscopy (SEM) images of Sb_2_Se_3_ thin films were measured using a Zeiss Gemini 450 SEM. X-ray diffraction (XRD) was obtained using the Rigaku Smartlab diffractometer. The Sb_2_Se_3_ and Au reference cards were obtained from the Cambridge Crystallographic Data Centre (CCDC) database.

### 3.5 TEM and FIB sample preparation

TEM lamellae were prepared by FIB (Helios 5 UX, Thermo Fischer Scientific) using AutoTEM 5 (Thermo Fischer Scientific). Carbon deposition was used to protect the surface. The chunk milling and lamellae thinning were done at 30 kV with an FIB current from 20 nA to 90 pA. Then the lamellae were polished at 5 kV and finished at 2 kV. TEM characterisation was performed on a TEM (Talos F200X, Thermo Fischer Scientific) operating at 200 kV. EDS mapping was acquired by using quadrant EDS detectors (Super-X, Thermos Fisher Scientific, the Netherlands) in STEM mode.

### 3.6 XPS

Synchrotron XPS data (Fig. 3a and b) were acquired at the PHOENIX I (X07MB) beamline at the Swiss Light Source using an end station.^30^ The spectra were acquired in a high vacuum (>1 × 10^−5^ Pa) using a photon energy of 4000 eV and a pass energy of 100 eV. The energy scale was calibrated using an Au reference sample, setting the Au 4f_7/2_ line to 84.0 eV. XPS depth profiling was conducted using a Physical Electronics (PHI) Quantum 2000 X-ray photoelectron spectrometer featuring monochromatic Al-Ka radiation generated from an electron beam operated at 15 kV and 32.3 W. The energy scale of the instrument was calibrated using an Au reference sample. The analysis was conducted at 1 × 10^−6^ Pa, with an electron take-off angle of 45° and a pass energy of 23.50 eV. The neutraliser was set to automatic. In both cases, surface elemental concentrations were determined from the photoelectron spectra after Shirley background subtraction using the instrument-specific sensitivity factors for calculation. As necessary, the core level spectra were plotted to deconvolute spectra with contributions from multiple elements. An asymmetric line shape (GL 30) was assumed for the core level emissions with a separation of Δ*E* = 9.34 eV for the Sb 3d doublet.

### 3.7 KP-FM

An Asylum Research AFM (MFP-3D) was used to measure the work function of the samples. The probe used for the measurement was an AC240TM-R3. For calibration of the work function of the tip, a highly ordered pyrolytic graphite (HOPG) was used, with a reported work function of ∼4.6 eV.^31^ To achieve a fresh HOPG surface, a piece of scotch tape was used to pull off a few top layers of the graphite, exposing a fresh, clean surface for calibration. The HOPG used was purchased from MikroMasch (Grade: ZYA).

The open-source Gwyddion software package and the Asylum Research built-in software were used to analyse the AFM pictures further and determine the average work function of the surface.

### 3.8 ToF-SIMS

Secondary ion mass spectrometry measurements were conducted on a ToF-SIMS.5 from IONTOF GmbH, Germany. The instrument was operated in spectroscopy mode, and Bi^+^ primary ions with an energy of 25 keV were used to analyse an area of 500 × 500 μm^2^. Sputtering was carried out with caesium ions with an energy of 500 V. The sputtering time was correlated with sputter depth by measuring the sputtered area with a Dektak Stylus Profilometer (Bruker, Germany).

### 3.9 Computational

Spin-polarized DFT-MD simulation was performed in the Born–Oppenheimer framework employing the CP2K program package on (001) Sb_2_Se_3_ at the interface with 16 Cu atoms.^32,33^ 8 Cu atoms are initially placed at around 3 Å distance from the (001) surface, and 8 Cu atoms are initially placed at the height of the (001) surface. The (001) Sb_2_Se_3_ slab consists of 240 atoms, where each Sb surface atom is terminated by S–H (some S–H are bridging two surface Sb atoms), and each Se atom is terminated by hydrogen atoms, as shown in Fig. 5a side view and in Fig. S14a† top view. The simulation box dimensions are *x* = 23.24 Å, *y* = 23.54 Å and *z* = 40.0 Å. Periodic boundary conditions (PBCs) are applied in all 3-directions of space. A vacuum of around 15 Å has been inserted to separate the periodic *z*-replicas. The atoms were described by GTH pseudopotentials. The Perdew–Burke–Ernzerhof (PBE) exchange–correlation function was employed in agreement with previous works, which have shown a good description of the properties of Sb_2_Se_3_.^19,34–36^ The DZVP-MOLOPT-SR basis set and a 400 Ry plane wave basis set for all atoms have been used, being a good compromise between computational cost and accuracy.^37^ Grimme's D3 dispersion correction was employed.^19,38,39^ The DFT-MD simulation was performed for ∼40 ps in the *NVT* ensemble at a temperature of 150 °C (Nosé–Hoover chain thermostat) and adopted the Velocity-Verlet algorithm with a time step of 0.5 fs.^40,41^ A uniform background charge and the Ewald summation for electrostatics take care of the total charge of the simulation box whenever necessary, as a standard procedure in DFT-MD simulations. The PBE functional was supplemented with the Hubbard *U* parameter in order to overcome the self-interaction error in the exchange and correlation of the over delocalized d- and f-orbitals (and the consequent underestimation of the Sb_2_Se_3_ band gap).^42–45^ Hubbard parameter *U* is known as the on-site Coulomb interaction energy.^45^*U* parameters have been calculated for Sb^3+^ and Se^2−^ atoms in order to reproduce the experimental band gap energy of around 1.2 eV of Sb_2_Se_3_. Values of *U* = 3 eV, *U* = 2 eV have been calculated and adopted for Sb^3+^ and Se^2−^ atoms, respectively. See ESI and Fig. S14 and S15† for modelling of the bulk crystalline structure of Sb_2_Se_3_ and for bulk structural, electronic and mechanical properties calculations.

## 4. Conclusions

This study demonstrates enhanced photovoltage and photocurrent improvements through low-cost and room-temperature surface modification treatments. Overall, the etching treatment with (NH_4_)_2_S + CuCl_2_ on the surface of the Sb_2_Se_3_ in substrate configuration improved the onset potential by 57% and the current density by 38%. This progressive improvement is due to the removal of the surface Sb_2_O_3_ layer on the surface after the etching treatment. This is firmly evidenced by XPS. This layer appears to cause an unfavourable surface band bending, causing surface recombination. Further improvement was also observed after CuCl_2_ solution treatment due to the binding of Cu to surface Se atoms which passivate the surface defects present on the surface of the material as a result of dangling bonds. The binding of Cu can be seen through ToF-SIMS and is supported by DFT calculations. As the stability, the light dependency and therefore the performance of the devices depends on the catalyst, further investigation can be done to address the loss of the catalyst itself and improve its durability. Despite the considerable improvement in the photovoltage *via* these simple surface treatments, there is still a significant loss of photovoltage due to bulk defects such as cation–anion antisite defects.^46^ Therefore, a better understanding and screening of other metal halides and nitrates, combined with our previously studied bulk passivation methods,^47^ could pave the way towards improved performance in Sb_2_Se_3_ photocathodes.

## Author contributions

S. D. T. and P. A. designed the experiment. W. Y. provided supervision and advice. P. A. fabricated and tested all the devices. F. C. undertook the DFT calculations. T. M. carried out the KP-FM and light intensity dependant measurements. R. C. and P. Z. performed the ToF-SIMS and FIB-TEM measurements, respectively. Z. N. operated and measured the XPS end station at the Swiss light source (SLS). S. L. provided supervision for the DFT calculations.

## Conflicts of interest

There are no conflicts to declare.

## Supplementary Material

TA-011-D3TA00554B-s001
