# Supplementary material for: Solution phase treatments of Sb2Se3 heterojunction photocathodes for improved water splitting performance
Source: J Mater Chem A Mater. 2023 Mar 21;11(15):8277–84. doi: 10.1039/d3ta00554b (PMC10088359; doi:10.1039/d3ta00554b)
Supplement: TA-011-D3TA00554B-s001 [file TA-011-D3TA00554B-s001.pdf]

Electronic Supplementary Material (ESI) for Journal of Materials Chemistry A.
This journal is © The Royal Society of Chemistry 2023

Supporting Information

Pardis Adams[a], Fabrizio Creazzo[a], Thomas Moehl[a], Rowena Crockett[b], Peng Zeng[c], Zbynek Novotny[d,e], Sandra Luber[a], Wooseok Yang*[f,g] and S. David Tilley*[a]

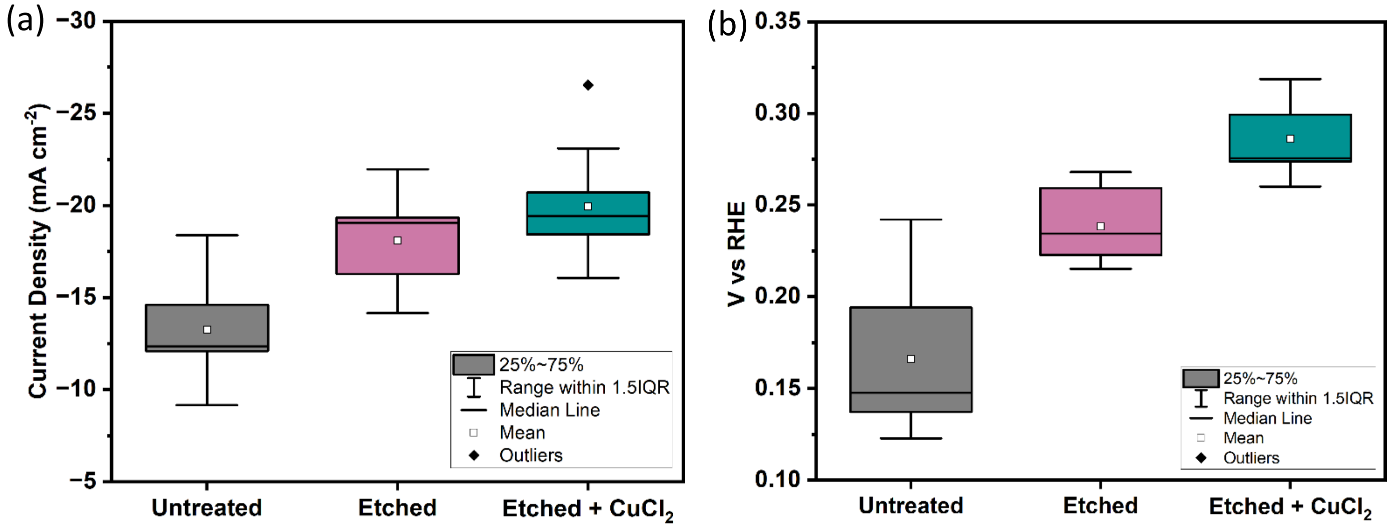

Fig. S1 Box plots of the onset potential and current density achieved from ten samples of each category.

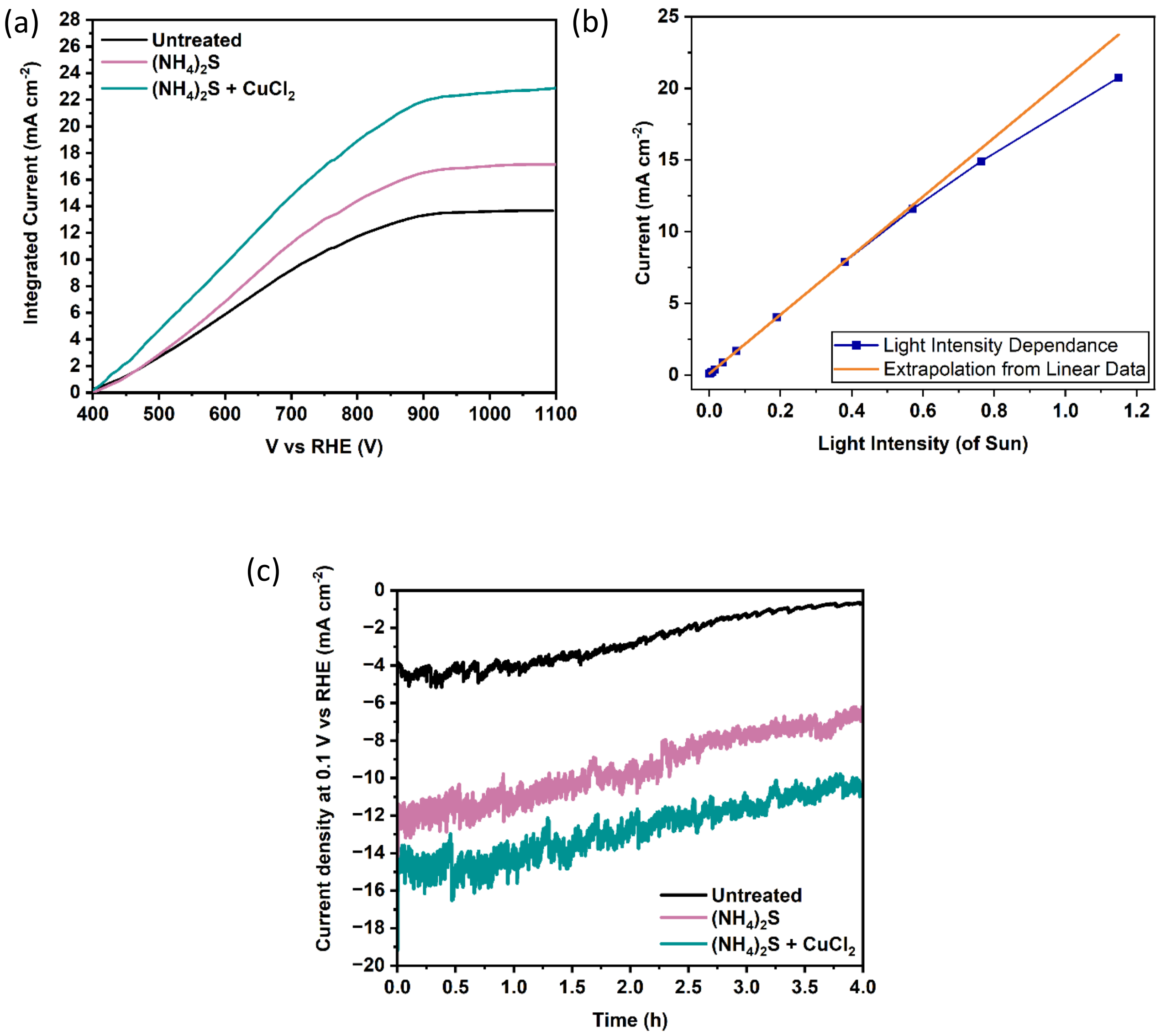

Fig. S2 (a) Integrated current (mA cm-2) vs wavelength obtained from IPCE data. (b) Light intensity dependence of a typical (NH4)2S + CuCl2 treated sample at different light intensities ranging from 0.1% to 120% sun (LED light source) and linear extrapolation of the data. (c) Current density vs time plots in 1 M H2SO4 at 0 V vs RHE at 1 sun illumination (100 mW cm-2) for 4 hours.

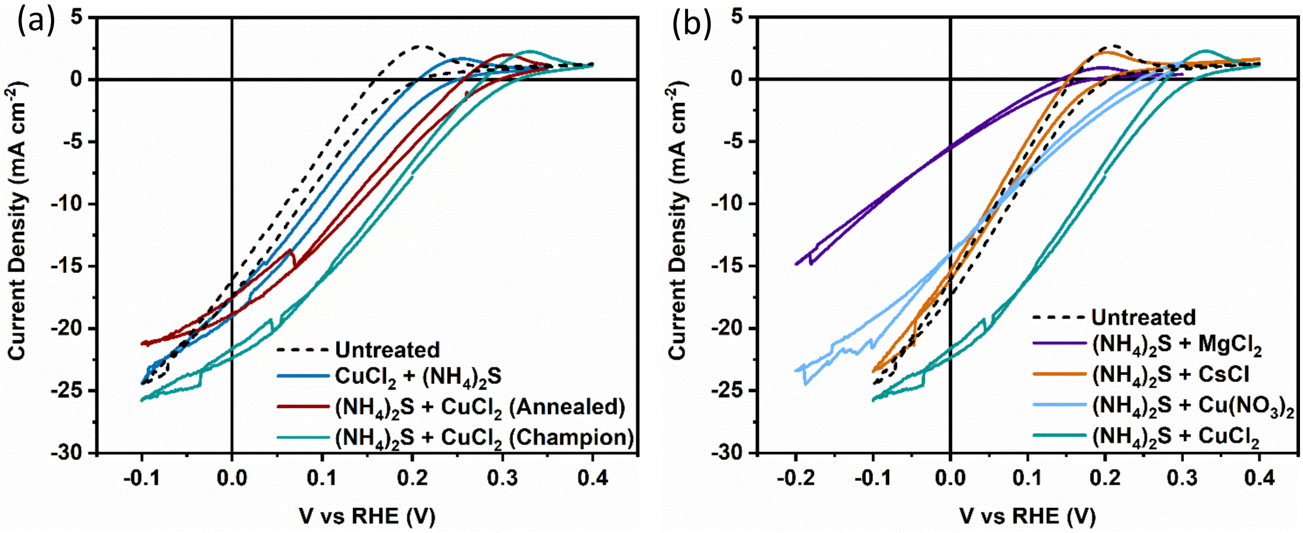

Fig. S3 (a) FTO/Ti/Au/Sb_2Se_3/TiO_2/Pt device with different treatments, swapped order of treatments and post-annealing at 200˚C for 20 minutes (b) FTO/Ti/Au/Sb_2Se_3/TiO_2/Pt device with different treatments including different copper and chloride sources, $MgCl_2$ and $Cu(NO_3)_2$ compared to champion and untreated devices.

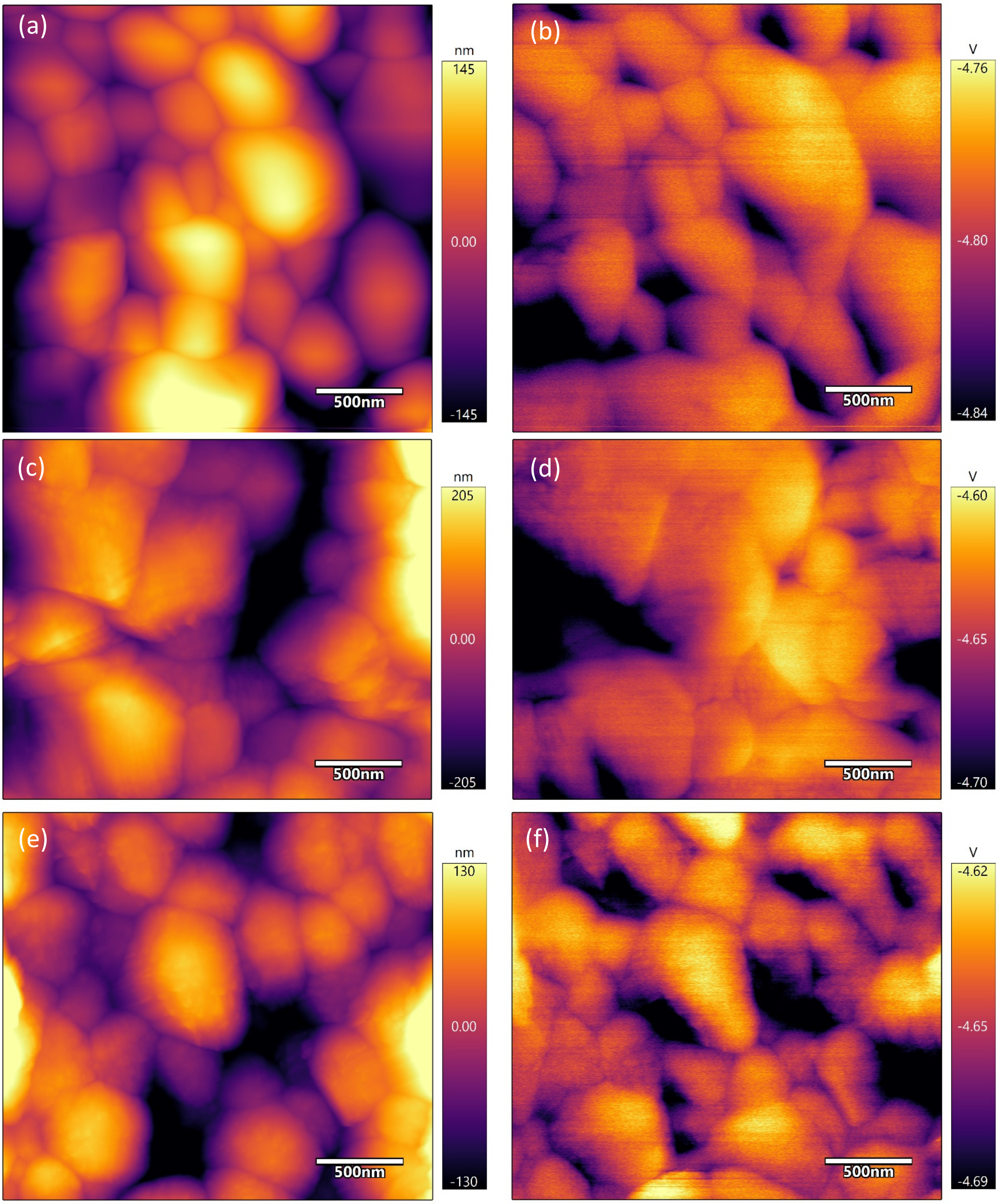

Fig. S4 AFM Topography and Kelvin Probe measurements: Topography of the unetched (a), the etched (c) and the etched and copper treated (e) sample. (b), (d) and (f) show the corresponding KPFM measurements.

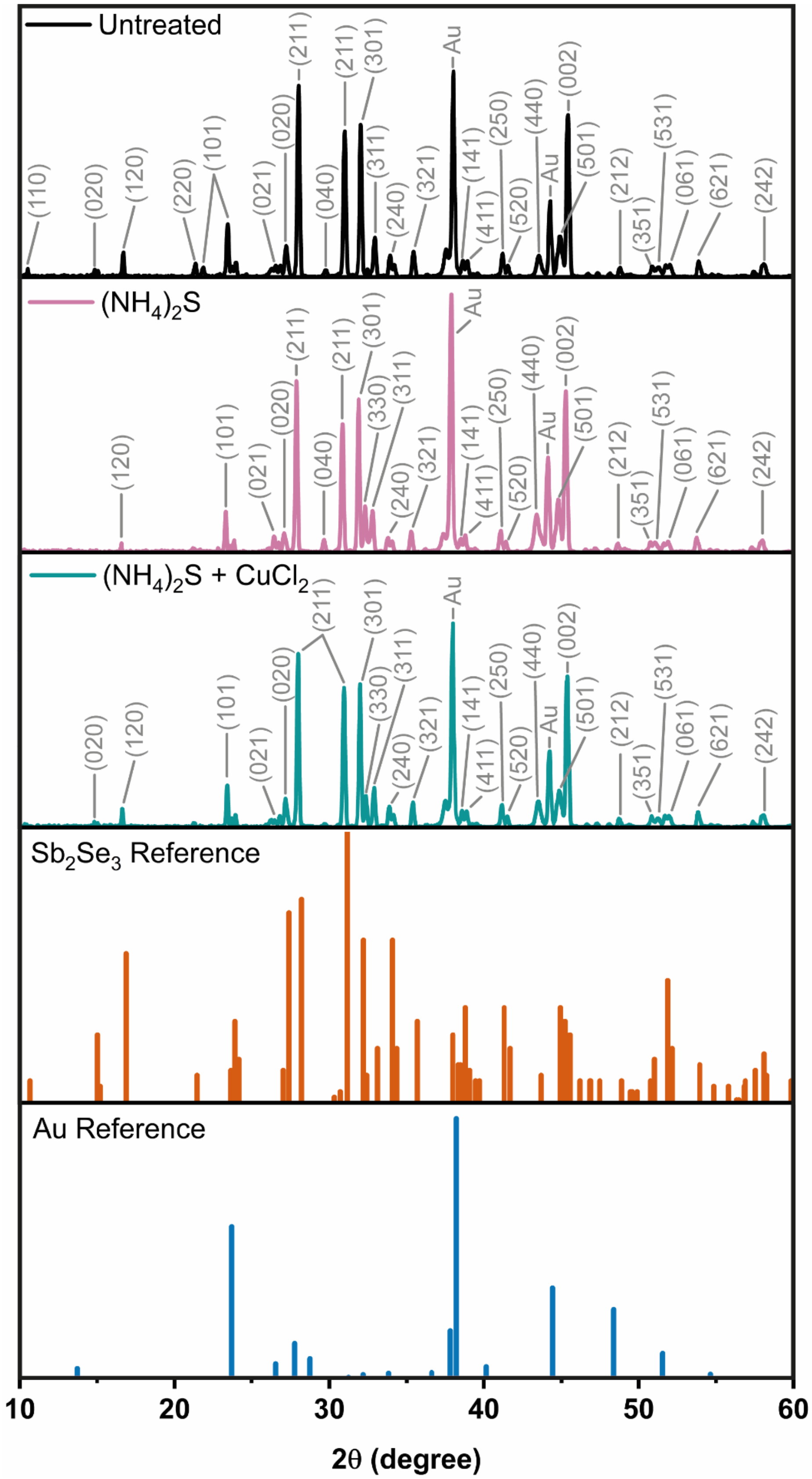

Fig. S5 XRD pattern of Sb_2Se_3 untreated, etched and etched + $CuCl_2$ compared to the orthorhombic phase of Sb_2Se_3 and Au reference cards.

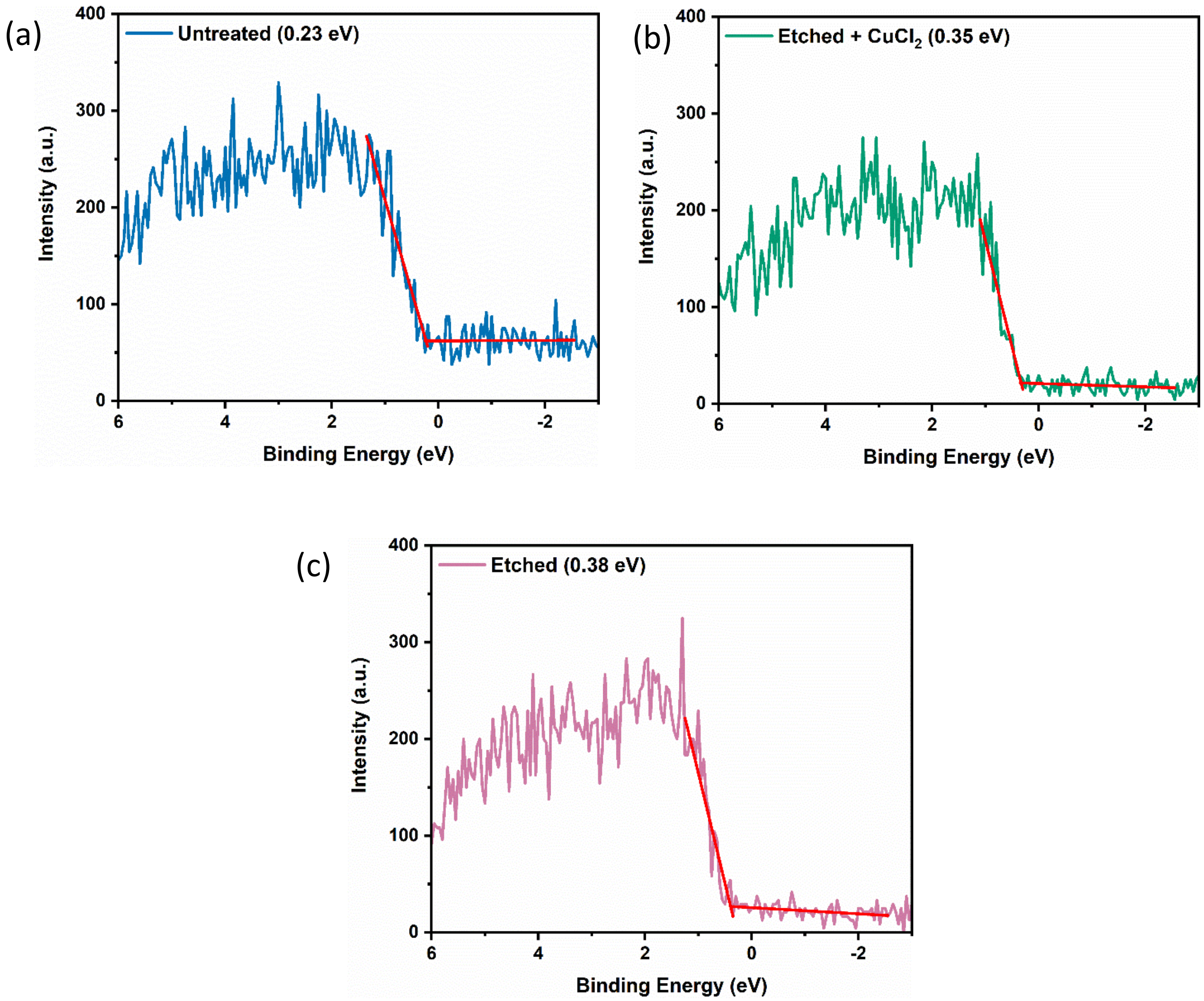

Fig. S6 Valence band maximum spectra of the untreated, etched and etched + $CuCl_2$ devices.

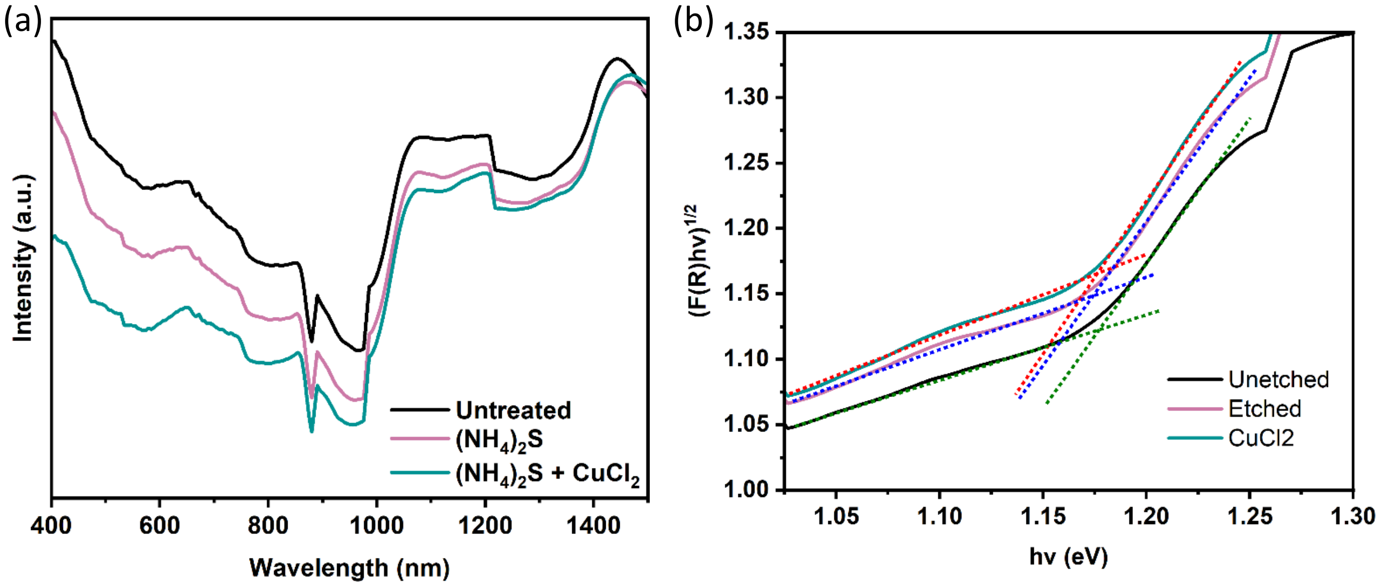

Fig. S7 (a) Diffuse reflectance spectra of the untreated, etched and etched + $CuCl_2$ devices. (b) Band gap of ~1.18 eV for all three samples was determined via the Kubelka-Munk (K-M) function.

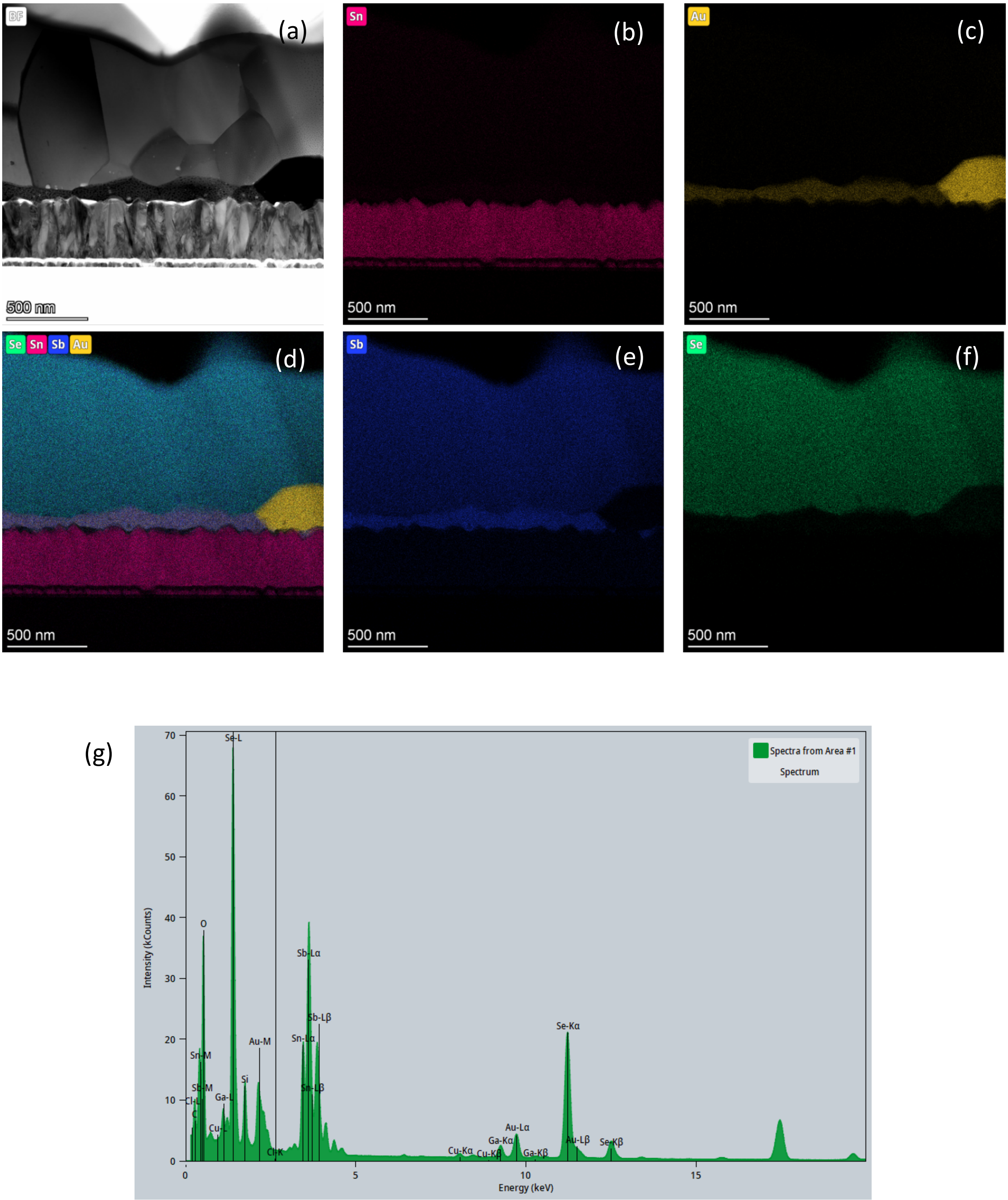

Fig. S8 (a-f) TEM cross-section and mapping of main elements. (g) the EDX spectrum of the $(NH_4)_2S$ + $CuCl_2$ sample, measured on a molybdenum grid.

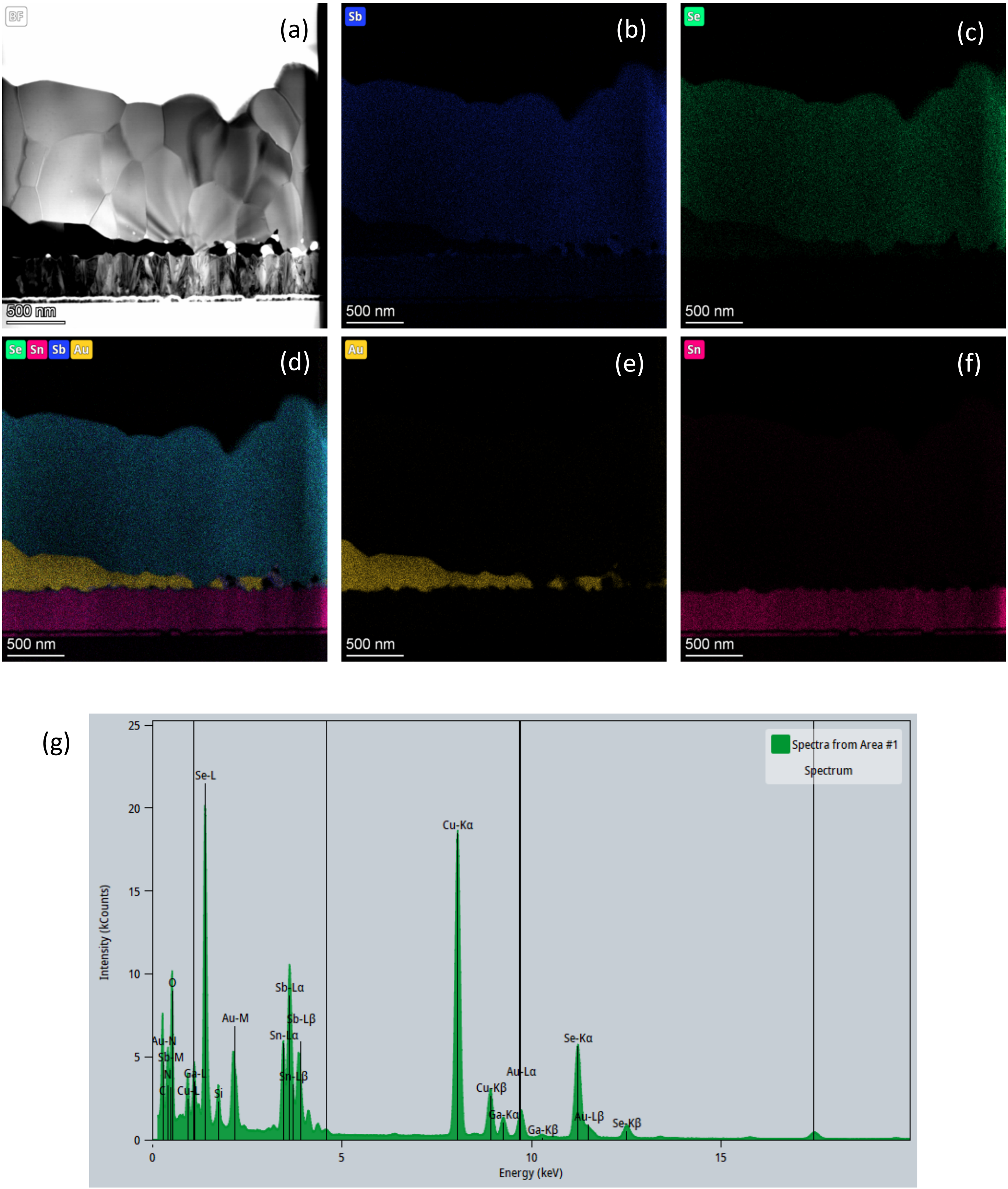

Fig. S9 (a-f) TEM cross-section and mapping of main elements. (g) the EDX spectrum of the $(NH_4)_2S$ etched sample, measured on a copper grid.

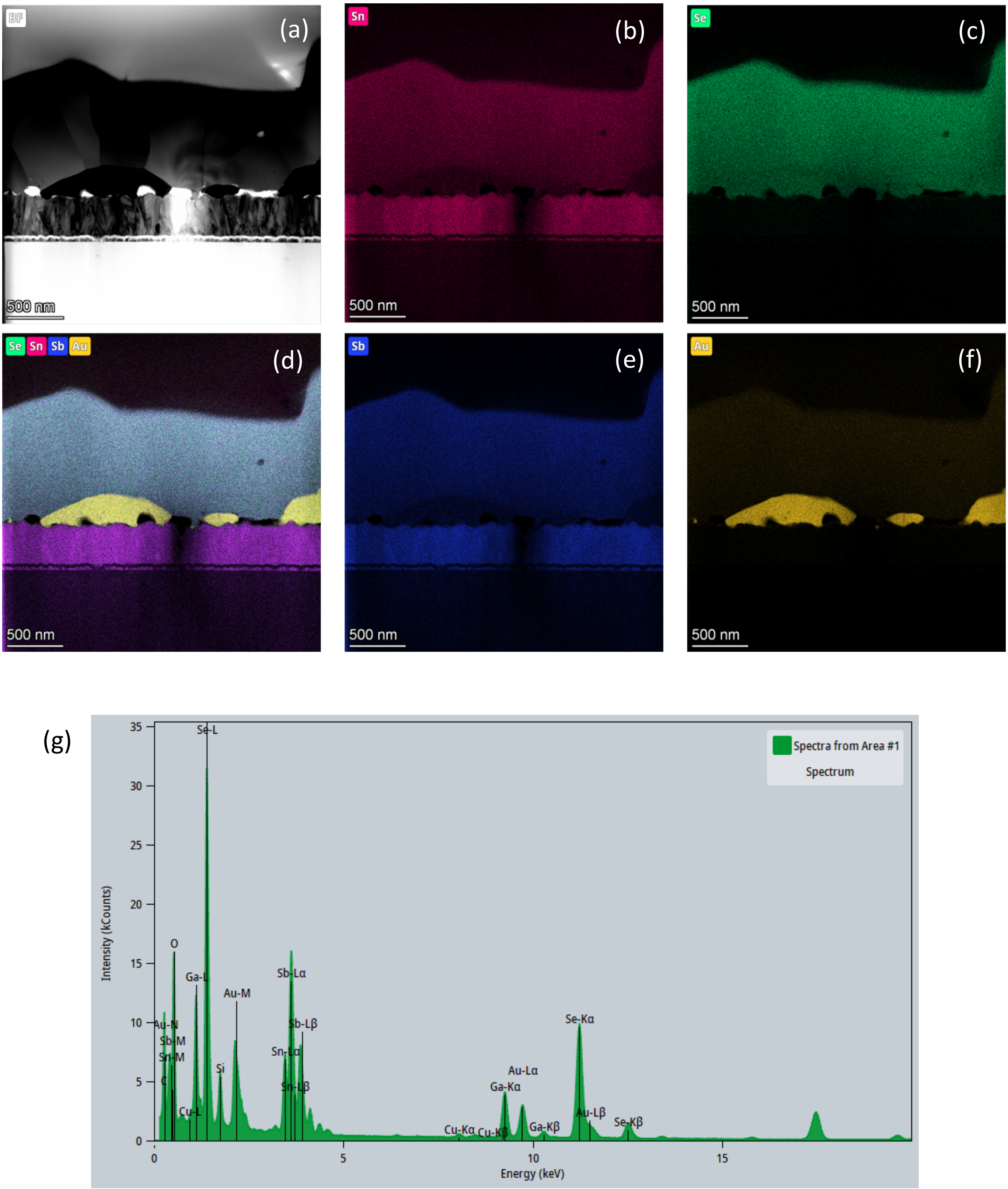

Fig. S10 (a-f) TEM cross-section and mapping of main elements. (g) the EDX spectrum of the untreated sample, measured on a molybdenum grid.

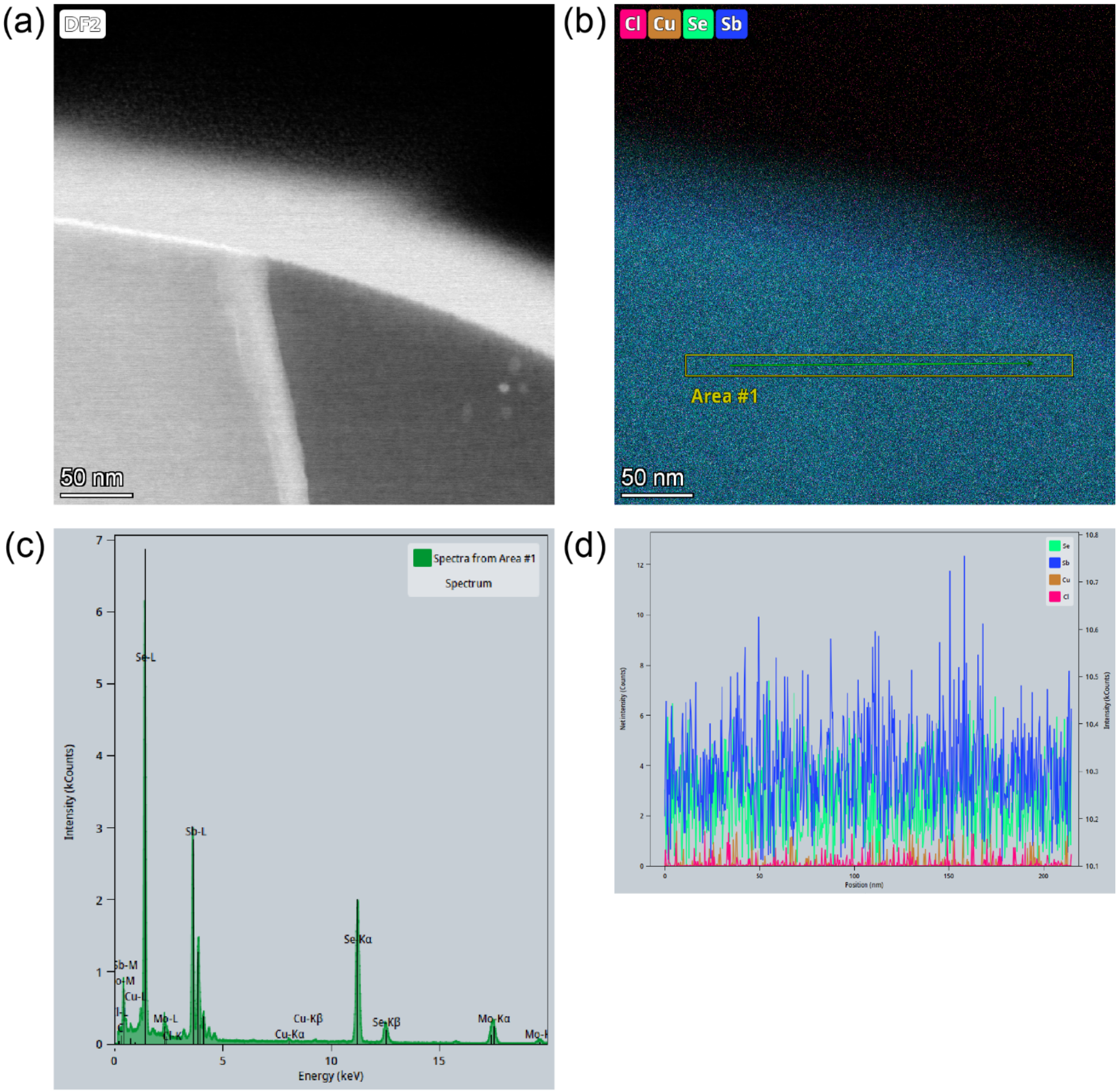

Fig. S11 TEM line scan of the grain boundary between two Sb_2Se_3 grains.

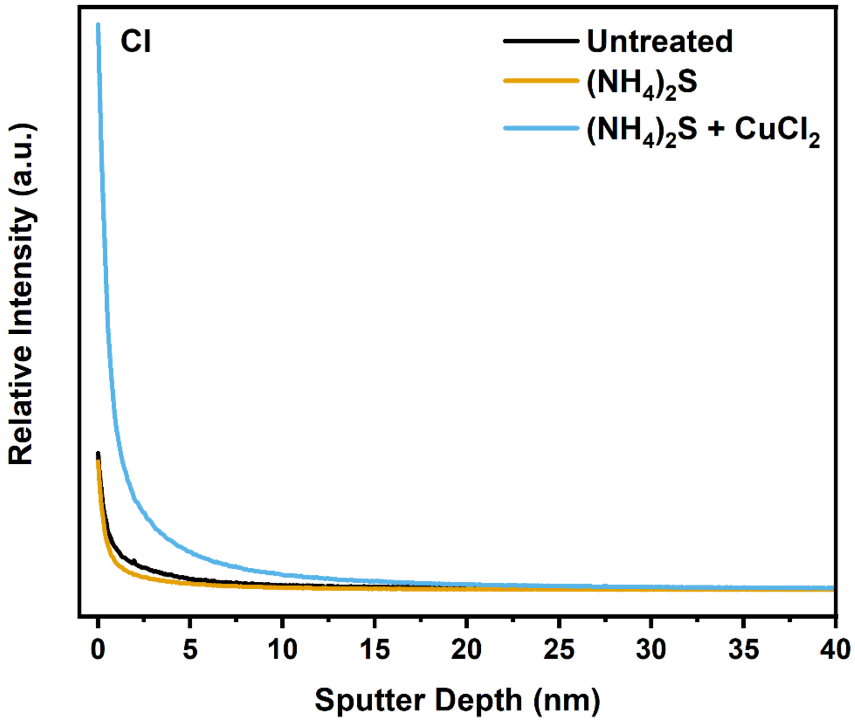

Fig. S12 Relative amount of chloride measured by ToF-SIMS depth profiling.

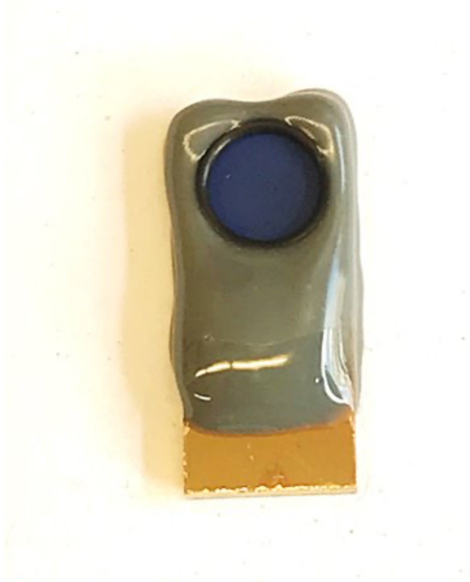

Fig. S13 A sample of Sb_2Se_3 with all overlayers and O-ring and epoxy for electrochemical measurement (2.5cm x 1cm, O-ring = 0.38 cm^2).

(a)

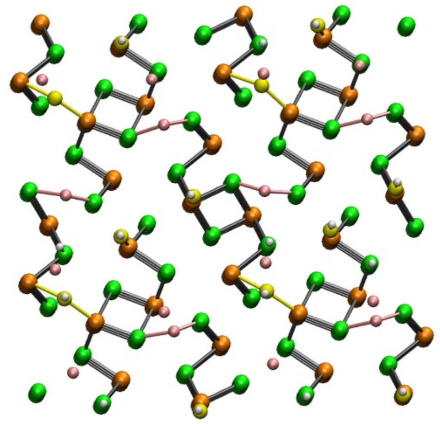

(b)

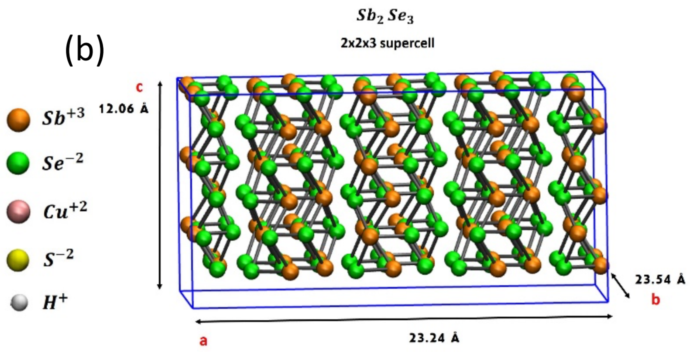

(c)

SYSTEM (n° of replicas)	Lattice Parameters of Unit Cell (a , b, c) (Å)
Unit (20 atoms)	9.09, 10.20, 5.44
141 (80 atoms)	11.35, 12.47, 4.04
222 (160 atoms)	11.51, 11.63, 4.58
223 (240 atoms)	11.62, 11.77, 4.02
333 (540 atoms)	11.36, 12.02, 4.06

(d)

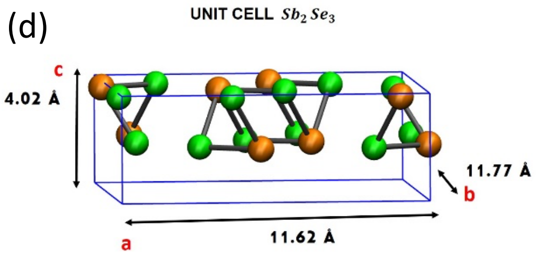

Fig. S14 (a) Top view of (001) Sb2Se3 at the interface with 16 Cu atoms. 8 Cu atoms are initially placed at around 3 Å distance from the (001) surface, and 8 Cu atoms are initially surface adsorbed by Se atoms at the (001) surface. The (001) Sb2Se3 slab consists of 240 atoms. (b) 2x2x3 supercell structure of bulk Sb2Se3 crystalline: 240 atoms in total. (c) Optimized lattice parameters for 141, 222, 223, and 333 supercell bulk structures. (d) Orthorhombic unit cell of bulk Sb2Se3 crystalline structure: 20 atoms in total, 8 Sb^{3+} and 12 Se^{2-} atoms in orange and green colour, respectively.

DFT calculations have been done at the Γ point of the Brillouin zone for the electronic representation; this imposes the use of a supercell, i.e., a certain number of replicas of the unit cell in 3D space. Geometry optimizations (atom positions and cell vectors) are performed on the unit cell and on 141 (80 atoms), 222 (160 atoms), 223 (240 atoms), and 333 (540 atoms) replica systems of the Sb_2Se_3 unit cell. Lattice parameters of the (2*x*2*x*3) supercell of Sb_2Se_3 are in good agreement with experimental results.[1,2] The total energy of the aforementioned supercell structures (141, 222, 223, 333) has been calculated as a function of volume by fitting the computed values to Murnaghan's equation of state.[3] In this way, a bulk modulus of around 30 GPa for the (223) Sb_2Se_3 supercell has been computed in good agreement with experimental results.[4,5]

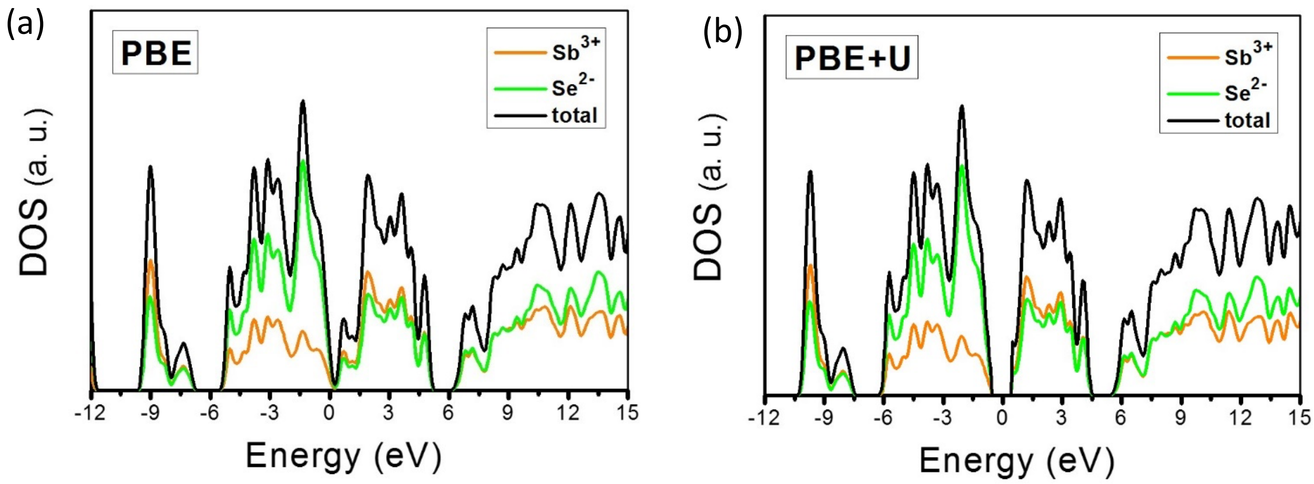

Fig. S15 Density of states (DOS) for the (223) Sb_2Se_3 bulk supercell. PBE (a) and PBE+U (b) calculations. U=3 eV, U= 2 eV for Sb^{3+} and Se^{2-} atoms, respectively. The Fermi energy level is set to 0.

The PBE+U calculation in Fig. S11-b shows that by adopting U=3 eV and U= 2 eV for Sb^{3+} and Se^{2-} atoms, it is possible to reproduce the experimental band-gap energy of around 1.2 eV for bulk Sb_2Se_3. No band gap is present without U correction; see Fig. S11-a.

References

1 G. P. Voutsas, A. G. Papazoglou, P. J. Rentzeperis and D. Siapkas, *Z Kristallogr Cryst Mater*, 1985, **171**, 261–268.

2 A. Kyono, A. Hayakawa and M. Horiki, *Phys Chem Miner*, 2015, **42**, 475–490.

3 M. M. Masayoshi Mikami, S. N. Shinichiro Nakamura, O. K. Osamu Kitao, H. A. Hironori Arakawa and X. G. Xavier Gonze, *Jpn J Appl Phys*, 2000, **39**, 847–850.

4 P. P. Kong, F. Sun, L. Y. Xing, J. Zhu, S. J. Zhang, W. M. Li, Q. Q. Liu, X. C. Wang, S. M. Feng, X. H. Yu, J. L. Zhu, R. C. Yu, W. G. Yang, G. Y. Shen, Y. S. Zhao, R. Ahuja, H. K. Mao and C. Q. Jin, *Sci Rep*, 2014, **4**, 6679.

5 I. Efthimiopoulos, J. Zhang, M. Kucway, C. Park, R. C. Ewing and Y. Wang, *Sci Rep*, 2013, **3**, 2665.
